# Supplementary material for: Dependence of Incidence Angle and Flux Density in the Damage Effect of Atomic Oxygen on Kapton Film
Source: Polymers (Basel). 2022 Dec 12;14(24):5444. doi: 10.3390/polym14245444 (PMC9781240; doi:10.3390/polym14245444)
Supplement: Supplementary file 1 [file polymers-14-05444-s001.zip › polymers-2063888-supplementary.pdf]

Supplementary Materials:

Title: Dependence of Incidence Angle and Flux Density in the Damage Effect of Atomic Oxygen on Kapton Film

1. Reflection efficiency calculation of neutral targets for the ground-based test device

The atomic oxygen flux density of the ground-based device is determined by both the target current (ion current) and the reflection efficiency of the target, as shown in Equation (1).

$$\begin{cases} F_i = \frac{I_i}{eA_C} \\ F_{AO} = \eta F_i \end{cases} \quad (1)$$

where F_{AO} is the atomic oxygen flux density, atoms cm^{-2}·s^{-1}; F_i is the ion flux density, ions cm^{-2}·s^{-1}; η is the neutral target reflection efficiency; I_i is the target current, A; e is the electron charge, 1.6×10^{-19} C; A_C is the neutral target collecting pole area, 36π cm^2.

An alternative method to calculate the atomic oxygen flux density is to use the mass loss calculation of a standard Kapton material under vertically incident atomic oxygen irradiation, as shown in Equation (2).

$$F_{AO} = \frac{\Delta m}{A \cdot E_y \cdot \rho \cdot t} \quad (2)$$

where F_{AO} is the atomic oxygen flux density, atoms cm^{-2}·s^{-1}; Δm is the mass loss, g; A is the surface area of the sample, 4 cm^2; E_y is the Kapton erosion yield, 3×10^{-24} cm^3/atom; ρ is the Kapton density, 1.4 g/cm^3; t is the irradiation time, 36000 s.

Kapton's atomic oxygen erosion yield is constant at low atomic oxygen flux density. Equation (1) (2) can be solved by the joint solution to obtain:

$$\eta = \frac{\Delta m}{I_i} \cdot \frac{eA_C}{A \cdot E_y \cdot \rho \cdot t} \quad (3)$$

Let$\frac{eA_C}{A \cdot E_y \cdot \rho \cdot t} = C_1$, C_1 is a constant, then:

$$\eta = C_1 \cdot \frac{\Delta m}{I_i} \quad (4)$$

In this thesis experiment, Kapton's mass loss data at 0° incidence angle showed a positive proportionality to the low target currents (0.25-1 A), normalized as shown in Figure S1. This indicated that $\frac{\Delta m}{I_i}$ is a certain value for this target current range. The erosion yield of atomic oxygen is essentially constant, the same as that for lower target currents (flux density), 3×10^{-24} cm^3/atom.

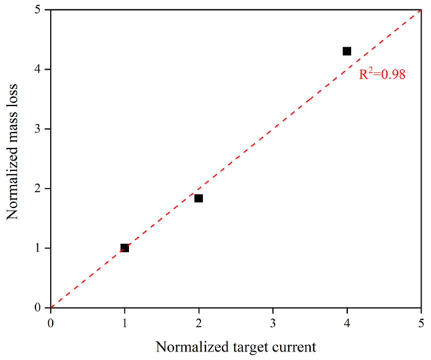

Figure S1. Normalized mass loss of the Kapton at different target currents

Substituting the atomic oxygen test data ($I_{i1} = 0.25A$, $\Delta m_1 = 0.0015g$; $I_{i2} = 0.5A$, $\Delta m_2 = 0.00275g$; $I_{i3} = 1A$, $\Delta m_3 = 0.00645g$) with an incidence angle of 0° into equation (4), we get: $\eta_1 = 0.180$, $\eta_2 = 0.165\eta$, $\eta_3 = 0.193$, and the average value of $\overline{\eta} = 0.179$, which means the reflection efficiency is about 18%.

2. Information on atomic oxygen calculation parameters in ReaxFF MD simulations

Table S1. Atomic Oxygen Parameters in ReaxFF MD Simulations.

Parameter / Job	Angel (°)	Vx (Å/fs)	Vz (Å/fs)	Dose rate (atoms/ps)	AO (atoms)	Time (ps)
1	0	0.0000	-0.0780	5	300	60
2	5	-0.0068	-0.0777	5	300	60
3	10	-0.0135	-0.0768	5	300	60
4	15	-0.0202	-0.0753	5	300	60
5	20	-0.0267	-0.0733	5	300	60
6	25	-0.0329	-0.0707	5	300	60
7	30	-0.0390	-0.0675	5	300	60
8	35	-0.0447	-0.0639	5	300	60
9	40	-0.0501	-0.0598	5	300	60
10	45	-0.0552	-0.0552	5	300	60
11	50	-0.0598	-0.0502	5	300	60
12	55	-0.0639	-0.0447	5	300	60
13	60	-0.0675	-0.0390	5	300	60
14	65	-0.0707	-0.0329	5	300	60
15	70	-0.0733	-0.0267	5	300	60
16	75	-0.0753	-0.0202	5	300	60
17	80	-0.0768	-0.0135	5	300	60
18	85	-0.0777	-0.0068	5	300	60
19	0	0.0000	-0.0780	5	150	30
20	0	0.0000	-0.0780	10	300	30
21	0	0.0000	-0.0780	20	600	30
22	0	0.0000	-0.0780	30	900	30
23	0	0.0000	-0.0780	40	1200	30
